# 619. Burn Unit Social Worker Environmental Scan Describes Workplace Burnout

**DOI:** 10.1093/jbcr/irag033.433

**Published:** 2026-04-06

**Authors:** Patricia Spitzer, Gemma Isaac

**Affiliations:** Vancouver General Hospital BC Burn Unit, Vancouver, BC; Vancouver General Hospital BC Burn Unit, Vancouver, BC

## Abstract

**Introduction:**

Burnout in healthcare is defined as emotional exhaustion, depersonalization, and reduced personal accomplishment from chronic occupational stress. While social work burnout has been broadly studied, research specific to burn unit social workers is limited. Burn units are high-intensity environments with complex psychosocial and logistical demands. This study examined burnout prevalence and features among burn unit social workers across North America.

**Methods:**

A survey measured allied health collaboration and burnout using Maslach Burnout Inventory (MBI) items plus open comments. Collaboration was assessed by availability and frequency of allied engagement; burnout by emotional exhaustion, disconnection, motivation loss, and accomplishment. Eligibility required current burn unit or burn center practice. Twenty-five participated.

**Results:**

Collaboration was reported “Often” (52%) or “Always”, M = 2.86 (scale 0–3). Allied availability was “Often” (43%) or “Always”, M = 2.62. Workload support was inconsistent: 48% “Sometimes” or “Never,” M = 2.05. Emotional exhaustion: 53% “Sometimes,” or “Often,” M = 2.32. Overwhelm: 58% “Sometimes,” or “Often,” M = 2.32. Low motivation: 47% “Sometimes,” or “Often,” M = 1.79. Stress impacting rapport: 63% “Sometimes,” or “Often,” M = 1.84. Disconnection: 58% “Sometimes,” or “Often,” M = 1.68. Despite risks, satisfaction remained high: 53% “Often, "or “Always,” M = 2.89. Accomplishment: 63% ‘Often,’ or "Always,” M = 3.00. Meaningful patient impact: 47% “Often,” or “Always,” M = 2.84. Seven provided comments noting reward and strain. Themes: emotional demands and self-care, burn care complexity, role recognition, training gaps, value of interdisciplinary collaboration. Longevity and fulfillment were emphasized despite stressors.

**Conclusions:**

Burn unit social workers reported strong collaboration and fulfillment but also frequent exhaustion, overwhelm, and limited workload support. Single-staffing and inconsistent resources elevate burnout risk.

**Applicability of Research to Practice:**

Recommendations: (1) develop burn-specific training; (2) enhance role recognition within teams; (3) implement structured self-care/prevention programs; (4) increase interdisciplinary training with nursing/psychology; (5) protect social work focus by limiting redeployment; (6) strengthen retention via mentorship and career development.

**Funding for the study:**

N/A.

